# *Oscheius myriophila* (Nematoda: Rhabditida) isolated in sugar cane soils in Mexico with potential to be used as entomopathogenic nematode

**DOI:** 10.21307/jofnem-2020-073

**Published:** 2020-07-28

**Authors:** Iveth del Rocio Castro-Ortega, Juan Manuel Caspeta-Mandujano, Ramón Suárez-Rodríguez, Guadalupe Peña-Chora, José Augusto Ramírez-Trujillo, Karina Cruz-Pérez, Iván Arenas Sosa, Víctor Manuel Hernández–Velázquez

**Affiliations:** 1 Universidad Autónoma del Estado de Morelos, Laboratorio de Control Biológico, Centro de Investigación en Biotecnología, Cuernavaca, México; 2 Laboratorio de Parasitología Animal, Facultad de Ciencias Biológicas, Av. Universidad No. 1001, Col. Chamilpa, Cuernavaca C.P. 6220, Morelos, México; 3 Laboratorio de Fisiología Molecular de Plantas, Centro de Investigación en Biotecnología, Cuernavaca, México; 4 Laboratorio de Parasitología Vegetal, Centro de Investigaciones Biológicas, Cuernavaca, México; 5 Universidad Nacional Autónoma de México Departamento de Medicina Molecular y Bioprocesos del IBT-UNAM. Av. Universidad 2001, Chamilpa, 62210 Cuernavaca, Morelos, México

**Keywords:** First report, México, *Oscheius myriophila*, sugar cane, virulence

## Abstract

A survey of entomopathogenic nematodes was conducted in sugar cane crops in a total of 14 soils, and positive results were obtained for strain MC5-2014 in the municipality of Tepalcingo, Morelos, in soil with a sandy loam texture and a pH of 6.4. Species determination was performed via morphological and morphometric techniques by searching for a tubular stoma with a swollen cylindrical pharyngeal body and a metacorpus in the basal part. The range of body length (*L*) was 750 to 1200 μm in females and 720 to 910 μm in males, while the corresponding maximum widths (*W*) of the body were 30 to 60 μm and 20 to 30 μm, respectively. Males exhibited bursa with a 1 + 1 + 3 + 3 distribution of papillae, and females exhibited a vulva located at the mid-body. For molecular identification, the ITS region of ribosomal DNA was used. Virulence tests (LC_50_) were conducted with *Galleria mellonella*, and a value of 4.732 was obtained for infective juveniles (IJs). Taking taxonomic and molecular characteristics into account, the isolate was determined to be *Oscheius myriophila*. The isolation of this strain represents the first geographic report of *O. myriophila* in Mexico, and it should be noted that the cultivation of sugar cane occurs with constant application of insecticides, herbicides, fungicides, and fertilizers as well as harvesting activities such as burning of the crop for harvest. The *O. myriophila* isolate has the potential to be used in the future as a method of biological control in our country.

Entomopathogenic nematodes (EPNs) exhibit a cosmopolitan distribution and have been isolated on five continents in different habitats around the world (Griffin et al., 1990; Hominik et al., 1996). In Mexico, EPNs, particularly species of the genera *Steinernema* and *Heterorhabditis*, have been isolated in the desert, forests, and arable areas (Lezama et al., 2001; Stock et al., 2009). Worldwide, the main studies and reported isolates have come from these genera. Recently, two new species of EPNs of the genus *Oscheius* have been reported: *Oscheius chongmingensis* and *O. carolinensis*, which are associated with the pathogenic bacteria of insects of the genus *Serratia* (Nguyen and Hunt, 2007; Abebe et al., 2010). The genus *Oscheius* was described as a sister taxon of *Rhabditis* (Dujardin, 1845) Sudhaus (1976). The genus *Oscheius* belongs to the Rhabditidae family and is divided into two groups: insectivora and dolichura. The species *O. carolinensis, O. amsactae, O. niazii,* and *O. siddiqii* of the insectivora group and one species of the dolichura group, *O*. *onirici*, have been recognized as EPNs (Tabassum and Shahina, 2010; Ye et al., 2010; Torrini et al., 2015). In 2017, it was reported that *O. myriophila* was associated with the European mole cricket, *Gryllotalpa gryllotalpa* (Orthoptera: Gryllotalpidae) (Zeynep et al., 2017). The objective of the present study was to isolate EPNs in sugar cane crops, and strain MC5-2014 was obtained, after which the *Serratia* bacterial strain was also isolated. This work provides the first geographic report of *Oscheius myriophila* in Mexico and the first global report of its entomopathogenic nature.

## Materials and methods

### Collection of soil samples

Soil samples were collected from 14 plots of sugar cane located in the state of Tepalcingo, Morelos in Mexico using the technique proposed by Stock et al. (1999), and a modified method described by Orozco et al. (2014). For soil sample collection, within a plot, samples were taken at five points, with three subsamples per site; for sampling, a grid of 3 m^2^ was established around the sugar cane plant, and 1 kg of soil was collected at a depth of approximately 15 cm; the soil samples were placed in plastic bags. The sites were sampled in September 2014.

Soil analysis was performed to determine soil physicochemical characteristics such as texture, pH, electrical conductivity, and % organic matter (MO) in the bioremediation laboratory of CEIB/UAEM. EPNs were extracted with the insect baiting technique as described by Orozco et al. (2014). Each soil sample was baited by placing 12 fifth-instar *Galleria mellonella* Linnaeus (Lepidoptera: Pyralidae) wax worms in speech boxes containing sampled soil, and they were stored at room temperature in the dark for a week. Subsequently, the boxes were checked daily until the wax worms began to show signs of infection, at which time the insects were removed from the boxes, rinsed with water and stored in modified white traps (Orozco et al., 2014) until the EPNs emerged. After emergence, the EPNs were collected and stored in 50 mL Corning^®^ cell culture flasks at 12 °C.

### Nematode identification

#### Morphological and morphometric characterization

Morphological and morphometric identification was performed using an optical microscope equipped with a micrometric eyepiece. The nematodes were extracted through the dissection of wax worm larvae, and the IJs were collected from the white trap according to the method proposed by Nguyen and Smart (1996).

#### Molecular characterization of nematodes

For identification with molecular techniques, three hundred IJs were frozen in liquid nitrogen and macerated with a plastic pestle. Their DNA was purified with a Puregene^®^ DNA Purification Kit following the manufacturer’s instructions. An 850 bp fragment corresponding to the D2D3 region of the 23S rDNA gene was PCR amplified with the primers pair D2F (5′CCTTAGTAACGGCGAGTGAAA-3′) and 536 (5′-CAGCTATCCTGAGGGAAAC-3′) (Nguyen et al., 2006). The reaction was conducted in a final volume of 50 µl containing each primer at 0.5 µM, 0.16 mM dNTPs, 1.5 mM MgCl_2_, 2 units of Taq DNA polymerase (Thermo Fisher Scientific Inc.) and 1X reaction buffer. The PCR product was verified by electrophoresis in a 1% agarose gel, and the bands were excised and purified using a QIAquick^®^ Gel Extraction kit (QIAGEN, Germany). The PCR conditions were 94 °C for 2 min initial denaturation; 37 cycles of 30 s of denaturation at 94 °C, 45 s of hybridization at 48 °C and extension at 72 °C for 90 s; with a final extension of 72 °C for 5 min. The PCR product was sent to the Biotechnology Institute of the National Autonomous University of Mexico for sequencing. The D2D3 region was analyzed using the BLAST program of the GenBank database of the National Institutes of Health.

#### Isolation and identification of the bacterial symbiont

For the extraction of symbiotic bacteria from MC5-2014, two methods were used. In the first method, 5000 freshly emerged IJs were obtained from the white trap, and were placed in Eppendorf tubes. The tubes were centrifuged at 1,000 revolutions per minute (rpm) for 7 min, and the supernatant was discarded. Thereafter, washes were conducted with 5% sodium hypochlorite, and three washes were performed with sterile distilled water (Lee and Stock, 2010). The IJ nematodes were macerated in a mortar and pestle for a period of 30 min and plated in solid Luria-Bertani (LB) and HCT (Bacto Tryptone (Difco) 5; Casamino acids (Difco) 2; pH adjusted to 7.5) media. After sterilization, culture medium consisting of KH_2_ PO_4_, 3.4 g/L; MgSO_4_.7H_2_ O, 0.012 g/L; MnSO_4_.4H_2_ O, 0.003 g/L; ZnSO_4_.7H_2_ O, 0.0028 g/L; Fe(SO_4_)3.7H_2_ O, 0.02 g/L; CaCl_2_.2H_2_ O, 0.147 g/L; and glucose, 3 g/L was used. For the second method of extraction from hemolymph, 10 larvae of *G*. *mellonella* at the 5th instar were infected with 10 IJs from a stock solution of MC5-2014 nematodes. After 96 hr, larvae with characteristics corresponding to death caused by EPNs were selected, and the outer parts of the larvae were disinfected with 70% alcohol for 10 min, followed by drying for 2 min inside a laminar flow hood (Woodring and Kaya, 1988).

Subsequently, a 0,3 ml insulin syringe whith a 31 × 6 mm needle were used to extract the hemolymph by inserting the needle between the 6th and 7th inter-segments of the larvae. An aliquot of the hemolymph was plated on solid LB and HCT culture media, and the plates were incubated for 24 hr at 27 °C. All colonies were sampled, and the cross-streak technique was used to obtain single colonies. The bacteria were identified by biochemical methods using Gram staining, motility tests, and assays of protease activity and lecithinase and antibiotic production as suggested by Boemare et al. (1997) and Akhurst (1980). For molecular identification of the isolated bacteria, the cells were incubated in LB liquid medium for 12 h at 30 °C, and DNA extraction was then carried out with the Easy-DNA™ kit following the instructions recommended by the manufacturer. Then, amplification of a partial 16S rRNA region (600 bp) was performed by PCR using the primer 63f (5′-CAG GCC TAA CAC ATG CAA GTC-3′) (Nübel et al., 1996). The PCR conditions were 95 °C for 3 min initial denaturation; 37 cycles of 35 s of denaturation at 95 °C, 42 s of hybridization at 59 °C and extension at 72 °C for 1 min 30 s; with a final extension of 72 °C for 5 min. The PCR products were identified at the Biotechnology Institute of the National Autonomous University of Mexico with the primer 63 f. The sequences were analyzed using the BLAST program of the GenBank database, National Institutes of Health.

#### Pathogenicity of the symbiont bacterium in Galleria mellonella

Pathogenicity bioassays of bacterial strain MC5-2014 were carried out in 10 larvae of the 5th stage of *Galleria mellonella,* and the treatments were carried out in triplicate. The bacterial isolates were cultured in solid LB medium for 18 hr at 27 ± 2 °C. The surfaces of the larvae were previously disinfected with 70% ethanol and inoculated by injection using 10 µl of the bacterial suspension at a concentration of 1 × 10^5^ cells. The bacterial concentration was obtained via volumetric quantification using a Neubauer chamber.

Strain Bar 86 of *S. marcescens* was used as a positive control, while the *Escherichia coli* ATCC strain and distilled water were used as negative controls. The injected larvae were placed in 60 × 15 mm Petri dishes with a meridian diet (bee honey 97.5 ml, glycerol 120 ml, wheat bran (sterile) 37.5 ml, rice cereal 300 g, yeast 75 g). The pathogenicity and the changes in the coloration of the cuticle were evaluated at 24 and 48 hr. The results were subjected to analysis of variance (ANOVA) and the Tukey test at the 0.05 probability. The SAS statistical package was applied.

#### Virulence of the bacteria

The virulence bioassays of the bacteria isolated from the MC5-2014 strain were performed on 30 larvae of *G*. *mellonella* in the 5th instar, and the treatment was carried out in triplicate. Bacterial strains were cultured in solid LB medium for 18 hr at 28 ± 2 °C prior to quantification with the help of a Neubauer camera; volumetric calculations were performed and verified, and four concentrations were used to determine virulence for *G*. *mellonella* (10^5^, 10^3^, 50, and 5 cells). The surfaces of the larvae were previously disinfected with 70% ethanol. The same positive and negative controls mentioned above were used. The larvae were placed in 60 × 15 mm Petri dishes with sterile filter paper with a meridian diet for *G. mellonella*. The percentage of mortality and the change in the coloration of the cuticle were evaluated at 24 and 48 hr. Virulence (LC_50_) was determined by PROBIT analysis using the POLO PLUS version 1.0 program (LeORa Software LLC).

#### Virulence of EPNs

Virulence was determined using 6, 10, 14, 18, or 20 infected juveniles (IJs) plus the control, which contained only sterile distilled water. Virulence was determined by obtaining the average lethal concentration (LC_50_) via PROBIT analysis using the POLO PLUS version 1.0 program. (LeOra Software LLC).

## Results and discussion

### Isolation of EPN in sugar cane soils

Among the 14 soil samples taken from different locations in the state of Morelos, Mexico, positive results were found in the municipality of Teplacingo, which has an altitude of 1100 mm, a warm subhumid climate, an average annual temperature of 24 °C and a minimum temperature of 14 °C, from a site with sandy loam soil with a pH of 6.4 and an organic material (OM) percentage of 2.64%. We obtained the MC5-2014 isolate from sugar cane crops, and there have been reports of positive isolation in sugar cane cultures of *O. maqbooli* and *O. sacchari*. in Pakistan (Tabassum and Shahina, 2002; Tabassum et al., 2016). Our MC5-2014 isolate provides an additional isolate of nematodes belonging to the genus *Oscheius* from sugar cane, but it is the first member of this genus to be identified in Mexico.

### Nematode identification through morphological and morphometric characterization

#### Description

*Female*: Small-sized nematodes. Slender body 760 to 1160 µm long slightly curved ventrally after fixation. Cuticles are finely annulated with fine longitudinal striations. The head is continuous with the body contour, with six separate well-developed lips. Pore-like amphids on the lateral lips. Cylindrical pharyngeal body with cheilorhabdions that are not notable. The median bulb was absent. The metacorpus is swollen and pharyngeal. The isthmus is distinct, cylindrical, and slightly narrow. Nerve rings usually surround the mid portion of the isthmus. Excretory pores are conspicuously located at the level of the basal bulb. The excretion duct is circularized and curved anteriorly, then posteriorly. Vulva near the mid body and lips protruding.

*Male:* Similar to the female in general morphology except for a smaller size of 720 to 910 µm in length. The body is straight after fixation. Spicules are paired, separate, symmetrical, and slightly curved ventrally with hooked tips. Head of the spicules with a rounded anterior end, lamina expanded in their proximal part and velum prominent. Bursa leptoderan and eight genital papillae (rays) of different lengths are with a 1 + 1 + 3 + 3 arrangement. There were 3 precloacal papillae and 6 postcloacal papillae. Papillae 8 and 7 form 6 groups, and pairs 5, 4, and 3 form the second postcloacal group. Papillae two and one are separated near the base or back of the spicules. The spicules are paired and separate. They are slightly curved and have a triangular head and a rounded tip. The lengths of the spicules are between 30 and 80 µm. The gubernaculum is ventrally flattened, and it follows the contour of the spicules. The proximal tip is curved upward. The length of the gubernaculum is 20 to 30 µm.

*Habitat and locality*: soil around the roots of sugar cane (*Saccharum officinarum*) in Morelos, Mexico.

The male, female and IJ specimens were deposited in the Nacional Collection of Helminthes of the Biology Institute of the National Autonomous University of Mexico (CNHE/UNAM) with registration number 11085.

*Diagnosis: O. myriophila* belongs to the insectivora group and exhibits a cylindrical pharynx, a body with a leptoderan bursa and eight genital papillae of different lengthsarrangedin a 1 + 1 + 3 + 3 formation.

*O. sacchari* was isolated from sugar cane soil and belongs to the insectivora group. It presents similarity in the morphology of the anterior part that is a characteristic of the genus. *O. sacchari* is similar to *O. myriophila* in the body length of males (average: 843 vs 862 µm), but exhibits a different body width (average 61.6 vs 28 µm) and longer spicules (average: 41 vs 50 µm). Posterior part arrangement with the papillae in a 1 + 1 + 1 + 3 + 3. In females, shorter body lengths (average: 1607 vs 964.5 µm) and narrower body widths (average: 37 vs 88 µm) were observed.

*O. necromenus* is similar to *O. myriophila* in the body length of males (average: 843 vs 790 µm), but those of females differ (average: 964.6 vs 1179 µm) as does their body width (average 28-46 µm), and they exhibit longer spicules and a longer gubernaculum (average: 41 vs 40; 22 vs 15 µm, respectively). Detailed species comparisons for the genus of *Oscheius* are given in Table 1.

**Table 1.** Comparison of morphometrics with the MC5-2014 isolate.

Characteristic	*Rhabditis myriophila* Poinar, (1986)	*Oscheius myriophila* Tabassum et al. (2016)	*Oscheius myriophila* MC5-2014	*Oscheius sacchari* Tabassum et al. (2016)	*Oscheius rugaroensis* Zhang et al. (2012)	*Oscheius necromenus* Sudhaus and Schulte (1989)
Locality	California	Turkey	Mexico	Pakistan	China	Iran
Type host/habitat	*Oxidis gracilis*		Soil,	Soil,	Soil	
*Female*						
	–	–	964.5	1607	1042	1179
L	–	(972-1530)	(760-1160)	(1362-2015)	(920-1179)	
	(830-1500)					
	–	–	37	88	49.5	69
W	–	(52-100)	(30-60)	(72-125)	(39.8-58.2)	(54-90)
			554			
V	–	–	(410-760)	–	–	–
			52			
Eggs	–	–	(50-56)	–	–	–
*Male*						
	–	–	843	862	1396	790
L	(830-1470)	(841-1175)	(720-910)	(760-1390)	(1195-1692)	(671-950)
	–	–	28	61.6	62.4	46
W	(380-800)	(52-72)	(20-30)	(53-75)	(46.3-66.2)	(36-5)0
	–		41	50	49.2	40
Spicules	–	(27-39)	(30-40)	(47-55)	(35.2-60.9)	(34-44)
G	–	–	22	20.3	19.7	15
–	–	–	(20-30)	(18-22)	(19.9-26.5)	(12-23)

Note: L: total body length, W: maximum body width, V: vulva, and G: gubernaculum.

Based on the morphometric characteristics of males and females that are commonly used for the identification of nematodes, 10 females and 10 males were observed under a microscope (Nikon eclipse e200), and morphometric measurements were performed (Table 1). The maximum and minimum ranges of the body length were obtained: L, body width: W, spicules, distance from the part before the vulva, and length of eggs. The average values were calculated.

### Molecular identification

For molecular identification, an 832 bp fragment of the D2D3 region of the 28s ribosomal gene was amplified by PCR. The sequence of the MC5 2014 isolate was deposited in GenBank under accession number MK418537, showing 99% identity with DF5020 of *O. myriophila* isolated in the USA.

In Turkey (Zeynep et al., 2017), a nematode associated with *G. gryllotalpa* was isolated, and similar to our work, these authors used IJs for DNA extraction. They obtained 99% identity with *O. myriophila* Gg1 and an isolate from the USA (AY602176).

Notably, Torrini et al. (2015) reported a new species of EPN belonging to the genus *Oscheius* using the D2D3 region of 28s rDNA, as in our investigation.

### Bacterial symbiont isolation and identification

For the macerated extraction and the hemolymph extraction methods, the bacterial colonies were isolated at 48 and 72 h, grown on solid LB and HCT agar plates, and initially assigned as MC5-R based on red colonies. The biochemical tests showed that the bacterial strain MC5-R was Gram negative and was positive for motility tests, protease activity and lecithin activity. From the genomic DNA extraction product, a fragment of 600 bp was obtained. Once obtained, the PCR products were sent to the Institute of Biotechnology of UNAM (National Autonomous University of Mexico). The sequence of the isolated MC5-R was deposited in GenBank with the accession number MK463930. A BLAST search indicated 100% similarity with an MK463930 sequence and *S. marcescens* with accession number KM099142.1.

### Pathogenicity of the bacterium

The percentage of mortality in *G*. *mellonella* larvae of the 5th instar resulting from treatment with a concentration of 1 × 10^5^ was 100% for bacterial isolate MC5-R, 96.66% for *S. marcesns* Bar 86 and 0% for the *E*. *coli* ATCC and sterile distilled water negative controls.

### Virulence of the bacterium

**Figure 1:**
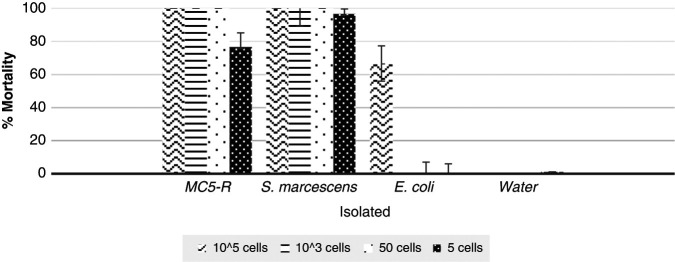
Mortality of *Galleria mellonella* larvae exposed to the MC5-R bacterium after 48 hours. 1: MC5-R, 2 S. marcescens, 3: E. coli, 4: water, means ± SD (range).

The percentage of mortality for *G*. *mellonella* larvae of the 5th instar resulting from treatment at a concentration of five cells per larvae was 76.66% for the bacterial isolate MC5-R and 0% and 1.11% for the *E*. *coli* ATCC and sterile distilled water negative controls, respectively (Figure 1). The result obtained with the bacterial strain MC5-R was an LD_50_ <10 cells injected in *G. mellonella* larvae at the 5th instar and the positive control *S. marcescens* Bar 86 showed an LD_50_ <10 cells, while that for the negative controls *E. coli* ATCC and distilled water was 0%.

### Virulence of the nematode MC5-2014 isolate

The mean lethal concentration was 4.732 IJ in *G. mellonella.* In EPN virulence evaluations in sugar cane borers, Grifaldo (Grifaldo et al., 2010) evaluated steinernematids in *D. saccharalis* in 2010 and obtained an LC_50_ of 3.7 IJ. The values obtained in this investigation were greater, and it should be taken into account that the behavior of the entomopathogens was different; this difference in virulence is influenced by different factors, such as the search strategy (Grewal et al., 1994) as well as their penetration and infectivity. In this study, *O. myriophila* was isolated, identified and evaluated, and solid evidence that it has potential to be used as an EPN is provided; it is worth mentioning that it is necessary to carry out an evaluation of the transmission of the bacteria to subsequent generations of nematodes (Dillman et al., 2012). The MC5-2014 isolate was virulent to *G. mellonella* larvae as quickly and efficiently as the isolated bacteria. An LC_50_ of 4.73 IJs/ml and an LD_50_ of <10 cells were obtained for the bacterium. The existing reports mention that species of the family Rhabditidae are pathogenic against pests with the aid of bacterial strains of the genus *Serratia,* which is the same bacterial species as the nematode MC5-2014 (Abebe et al., 2010; Ye et al., 2010; Torres-Barragan et al., 2011; Zhang et al., 2012).

Currently, species belonging to the genus *Oscheius* have been reported as EPNs, and the results obtained in our research highlight the potential of *O. myriophila* to be used in the future in biological control. However, to consider *O. myriophila* as an EPN, it is necessary to perform more evaluations on the type of association that exists with the bacteria isolated from this nematode.
